# Bioinformatics-Based Discovery of *CKLF*-Like *MARVEL* Transmembrane Member 5 as a Novel Biomarker for Breast Cancer

**DOI:** 10.3389/fcell.2019.00361

**Published:** 2020-01-09

**Authors:** Juan Zhou, Jian Lei, Jun Wang, Chen-Lu Lian, Li Hua, Zhen-Yu He, San-Gang Wu

**Affiliations:** ^1^Department of Obstetrics and Gynecology, The First Affiliated Hospital of Xiamen University, Teaching Hospital of Fujian Medical University, Xiamen, China; ^2^Department of Radiation Oncology, Cancer Hospital, The First Affiliated Hospital of Xiamen University, Teaching Hospital of Fujian Medical University, Xiamen, China; ^3^Department of Radiation Oncology, Sun Yat-sen University Cancer Center, State Key Laboratory of Oncology in South China, Collaborative Innovation Center of Cancer Medicine, Guangzhou, China

**Keywords:** DNA methylation, prognosis, biomarkers, disease progression, breast neoplasms

## Abstract

Chemokine-like factor (CKLF)-like *MARVEL* transmembrane members (*CMTMs*) represent a novel protein family linking the chemokine and transmembrane-4 superfamily families, which potentially play several roles in diverse physiological and pathological processes. The detailed functions and underlying molecular mechanisms of *CMTMs* remain elusive in breast cancer. Herein, we performed a comprehensive bioinformatic analysis to investigate the prognostic effect, potential functions, and biomolecular regulatory network of *CMTMs* in breast cancer. The mRNA expression level of *CMTM5*, in particular, was significantly downregulated in breast cancer; moreover, high mRNA expression level of *CMTM5* was significantly associated with better relapse-free survival. DNA promoter hypermethylation of *CMTM5* was negatively correlated with its mRNA expression levels. Furthermore, *CMTM5* strongly associated with pathway in MARVEL domains, chemotaxis, cytokines, transmembrane structures, and integral component of membrane. For example, genes related to MARVEL domains, transmembrane structures, and chemokines were significantly enriched. Our findings indicate that *CMTM5* can be used as a prognostic biomarker and potential therapeutic target for breast cancer.

## Introduction

Breast cancer is the most common malignancy in women; approximately 2 million new cases are annually diagnosed worldwide (Ferlay et al., 2015). Various molecular markers, including estrogen receptor (*ER*), progesterone receptor (*PR*), human epidermal growth factor receptor-2 (*HER2*), and *Ki67*, are widely used in the prognosis of breast cancer and to make treatment-related decisions (Untch et al., 2019). In recent decades, the early diagnostic techniques and clinical management of breast cancer have considerably improved (Merino Bonilla et al., 2017). However, distant metastasis remains an important risk factor related to outcomes in breast cancer, seriously affecting patient quality of life and contributing to a major socioeconomic burden. Therefore, there exists an urgent need to explore the mechanisms underlying breast cancer development and progression as well as to identify novel biomarkers to further improve its diagnosis, treatment, and prognosis.

In 2005, chemokine-like factor superfamily members 1–8 (*CKLFSF1–8*) were renamed *CKLF*-like *MARVEL* transmembrane domain-containing 1–8 (*CMTM1–8*). *CKLF* and *CMTM1*–8 represent a novel protein family linking the chemokine and transmembrane-4 superfamily (*TM4SF*) families, which potentially play several roles in diverse physiological and pathological processes (Han et al., 2003). *CKLF* and *CMTM1–4* are located in a gene cluster on 16q22.1, *CMTM6–8* are located in another gene cluster on chromosome 3p22, and *CMTM5* is located in 14q11.2 separately. *CMTM* family members play a key role in the immune system, tumorigenesis, and male reproductive system (Lu et al., 2016). *CMTM1–8* are known tumor suppressor genes (Bu et al., 2008; Li et al., 2011, 2015; Liu et al., 2013, 2015; Xie et al., 2014; Zhang et al., 2014, 2016; Gao et al., 2015; Xiao et al., 2015; Bei et al., 2017; Choi et al., 2018; Huang et al., 2019; Zhu et al., 2019); in addition, *CMTM6* is a key regulator of *PD-L1* in various human cancer cells. *PD-L1* relies on *CMTM6/4* to efficiently exert its inhibitory functions (Burr et al., 2017; Mezzadra et al., 2017). However, little remains known regarding the function of *CMTM* family members in breast cancer development and progression.

Herein, we performed a comprehensive bioinformatic analysis to investigate the prognostic effect and biomolecular regulatory network of *CMTMs* in breast cancer development and progression.

## Materials and Methods

### cBio Cancer Genomics Portal

We used the open-access cBio Cancer Genomics Portal (cBioPortal)^1^ to explore the relationship between *CMTMs* and genetic alteration frequency in 963 breast cancer samples (Cerami et al., 2012). Data from the Breast Invasive Carcinoma [The Cancer Genome Atlas (TCGA), provisional, 1108 samples] Project were used for analysis. GISTIC 2.0 was used to assess significant somatic copy-number alterations, such as deletions and amplifications (Mermel et al., 2011). The correlations among CMTM5 and related genes were also assessing with cBioPortal, and co-occurrence genes were defined as positive correlation, while genes that displayed mutual exclusivity were defined as negative correlation.

### Oncomine

Oncomine^2^ was employed to analyze the mRNA expression levels of *CMTMs* in various human cancers and corresponding normal tissues (Rhodes et al., 2004). In addition, the roles of *CMTM5* expression levels in response to breast cancer therapies were also investigate using Oncomine.

### UALCAN

UALCAN is an open-access, interactive web portal for analyzing cancer transcriptome data from the TCGA database^3^ (Chandrashekar et al., 2017). We used this portal to assess *CMTM* mRNA expression and DNA promoter methylation levels in breast cancer tissues and corresponding normal tissues.

### Kaplan–Meier Plotter

We used the Kaplan–Meier (KM) plotter to assess the potential prognostic effect of *CMTMs* on breast cancer^4^ (Lánczky et al., 2016). Survival curves (relapse-free survival, RFS) were plotted using the KM method and compared by the log-rank test.

### Methylation Modification Analyses

MEXPRESS was used to assess the relationship between *CMTM5* mRNA expression and DNA methylation levels in 871 invasive breast carcinoma samples (Koch et al., 2015). The relationship between *CMTM5* mRNA expression and DNA promoter methylation levels in 748 invasive breast carcinoma samples was also determined using MethHC, which is a database of DNA methylation and mRNA expression profiles in human cancers (Huang et al., 2015).

### Biomolecular Regulatory Network Analyses

We assessed the functional protein–protein interaction network for *CMTM5* using STRING^5^ with a confidence score of 0.4 (Szklarczyk et al., 2015). In addition, the Database for Annotation, Visualization and Integrated Discovery (DAVID) was used for gene ontology (GO) enrichment and Kyoto Encyclopedia of Genes and Genomes (KEGG) pathway analyses of *CMTM5*-related genes (Huang da et al., 2009a, b).

### Immunohistochemistry Data

We used the open-access Human Protein Atlas^6^ to analyze protein expression patterns of *CMTM5* in breast cancer. In general, gene expression patterns can be examined using this portal at the protein level using antibody-based protein profiling data. We used staining intensity to assess protein expression levels.

### Statistical Analyses

The patients clinical characteristic parameters were extracted from TCGA database. The correlation between clinical characteristic parameters and the expression of *CMTM5* in breast cancer was compared by one-way ANOVA test, and the results were expressed using mean ± standard deviation (SD). The statistical tests were performed using SPSS 22.0 (IBM Corp., Armonk, NY, United States). *P* values <0.05 were considered to indicate statistical significance.

## Results

### mRNA Expression Levels and Differences in Genetic Alteration Frequency of *CMTMs* in Human Cancers

**FIGURE 1:**
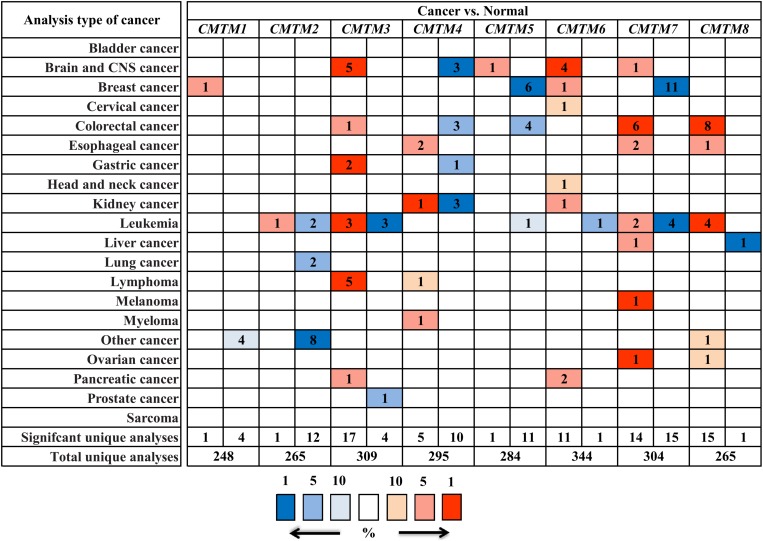
mRNA expression levels of *CMTM* family members in different human cancers and corresponding normal tissues from the Oncomine database.

To assess the role of *CMTMs* in human carcinogenesis, we used Oncomine to investigate mRNA expression levels of *CMTM* family members in various human cancers. The mRNA expression levels of *CMTM1*, *CMTM2*, *CMTM4*, and *CMTM5* were significantly downregulated whereas those of *CMTM3*, *CMTM6*, and *CMTM8* were noticeably upregulated in human tumors than in normal tissues. In addition, the mRNA expression of *CMTM7* was at a comparable level between human tumors and corresponding normal tissues (Figure 1). In breast cancer tissues, the mRNA expression levels of only *CMTM1*, *CMTM5*, *CMTM6*, and *CMTM7* were measured. The mRNA expression levels of *CMTM5* and *CMTM7* were significantly downregulated while those of *CMTM1* and *CMTM6* were significantly upregulated in breast cancer tissues than in corresponding normal tissues (Figure 1).

**FIGURE 2:**
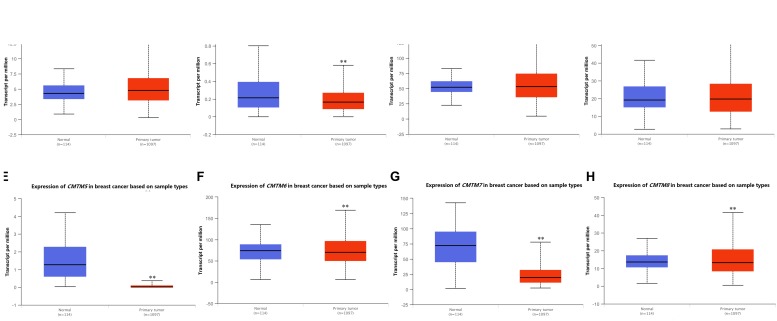
mRNA expression levels of *CMTM* family members in invasive breast carcinoma samples and corresponding normal tissues from the UALCAN database (^∗∗^*P* < 0.01). **(A)** *CMTM1*, **(B)** *CMTM2*, **(C)** *CMTM3*, **(D)** *CMTM4*, **(E)** *CMTM5*, **(F)** *CMTM6*, **(G)** *CMTM*7, and **(H)** *CMTM8.*

Next, we used UALCAN to confirm the mRNA expression levels of *CMTM* family members. We found that the expression levels of all *CMTM* family members were significantly dysregulated in breast cancer tissues. Among them, the mRNA expression levels of *CMTM5* [normal tissues vs. breast cancer tissues, 45.36-fold (*P* = 1.44*e*−08)] and *CMTM7* [normal tissues vs. breast cancer tissues, 3.72-fold (*P* < 1*e*−12)] were significantly downregulated, whereas those of *CMTM1* (1.11-fold, *P* = 8.41*e*−12) and *CMTM3* (1.02-fold, *P* = 3.43*e*−04) were only slightly upregulated and of *CMTM2* (1.28-fold, *P* = 1.15*e*−02), *CMTM6* (1.07-fold, *P* = 1.29*e*−02), and *CMTM8* (1.03-fold, *P* = 1.90*e*−05) were only slightly downregulated in breast cancer tissues than in corresponding normal tissues (Figure 2).

**FIGURE 3:**
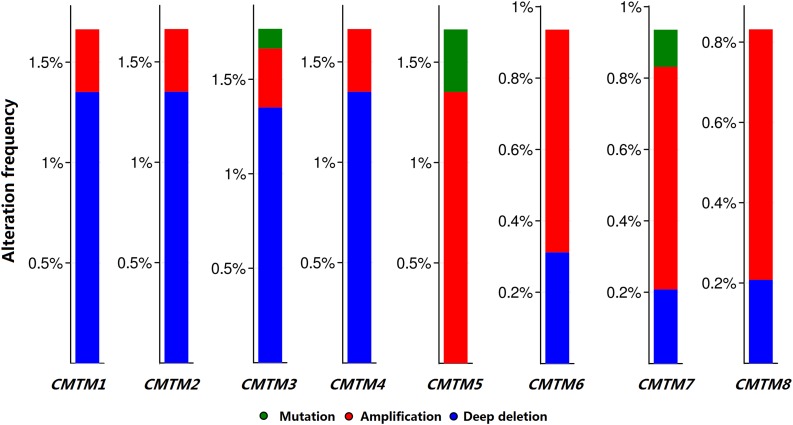
Genetic alteration frequency data of *CMTM* family members in breast cancer using cBioPortal.

Moreover, we analyzed the genetic alteration frequency data pertaining to *CMTM* family members in 963 breast cancer samples using cBioPortal. Alterations and amplifications were more likely to observe in case of *CMTM5*, and *CMTM1–4* were more likely to have a deep deletion (Figure 3). However, there were no significant differences between disease-free survival (*P* = 0.723) and overall survival (*P* = 0.904) in breast cancer patients with or without *CMTM5* alterations.

### Prognostic Effects of *CMTMs* on Breast Cancer

**FIGURE 4:**
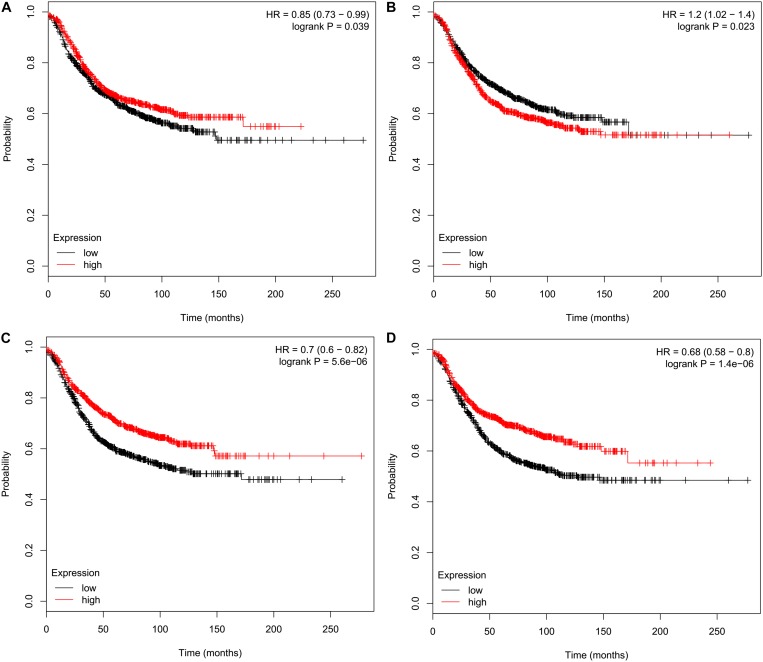
Relapse-free survival analysis of *CMTM* family members in breast cancer using the Kaplan–Meier plotter. **(A)** *CMTM3*, **(B)** *CMTM4*, **(C)** *CMTM5*, and **(D)** *CMTM7.*

We performed survival analyses to assess prognostic effects of *CMTMs* on breast cancer using the KM plotter. The obtained results indicated that high mRNA levels of *CMTM3* [hazard ratio (HR) 0.85, 95% confidence interval (CI) 0.73–0.99, *P* = 0.039] (Figure 4A), *CMTM5* (HR 0.70, 95% CI 0.60–0.82, *P* = 5.60*e*−06) (Figure 4C), and *CMTM7* (HR 0.68, 95% CI 0.58–0.80, *P* = 1.40*e*−06) (Figure 4D) were significantly related to better RFS; however, high mRNA levels of *CMTM4* (HR 1.2, 95% CI 1.02–1.40, *P* = 0.023) were significantly related to poor RFS (Figure 4B). In addition, mRNA levels of *CMTM1* (*P* = 0.510), *CMTM2* (*P* = 0.051), *CMTM6* (*P* = 0.970), and *CMTM8* (*P* = 0.910) were not correlated at all with RFS. These findings suggest that *CMTM5* has a higher prognostic effect on breast cancer than other *CMTM* family members; therefore, we selected *CMTM5* for further analyses.

### *CMTM5* mRNA Expression Levels and Prognostic Effect According to Clinicopathological Characteristics

**FIGURE 5:**
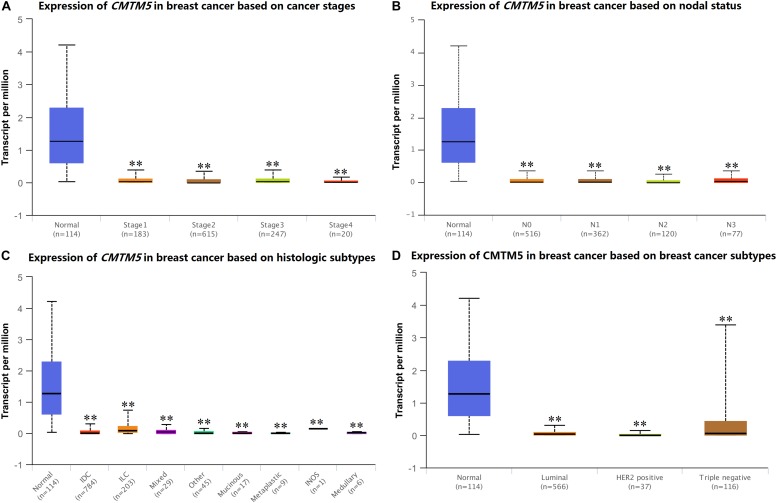
mRNA expression levels of *CMTM5* in breast cancer according to different clinicopathological characteristics from the UALCAN database (^∗∗^*P* < 0.01). **(A)** Tumor stage, **(B)** nodal stage, **(C)** histologic subtypes, and **(D)** breast cancer subtypes.

We used UALCAN to assess *CMTM5* mRNA expression levels in breast cancer according to clinicopathological characteristics. We found that the mRNA expression levels of *CMTM5* in breast cancer tissues were still significantly lower than in normal tissues after stratification by tumor stage, nodal stage, histologic subtypes, and breast cancer subtypes (Figures 5A–D).

After assessing the expression and prognosis of *CMTM5* in breast cancer, we further analyzed the correlation between patients clinical characteristics and the *CMTM5* expression levels. The patient clinical characteristic parameters have listed in Table 1. The levels of *CMTM5* expression were significantly associated with age (*P* = 0.016), *ER* status (*P* < 0.001), and *PR* status (*P* < 0.001). Patients with older age, and *ER* positive and PR positive disease had lower levels of *CMTM5* expression.

**TABLE 1:** The correlation between clinical characteristic parameters and the expression of CMTM5 in breast cancer.

**Variables**	**Number**	**Mean ± SD**	***P***
**Age (years)**			
<50	295	0.26 ± 1.34	0.016
≥50	1090	0.11 ± 0.71	
**Gender**			
Male	12	0.01 ± 0.01	0.585
Female	1078	0.15 ± 0.93	
**Histology (*n* = 1089)**			
Invasive ductal carcinoma	779	0.14 ± 0.83	0.286
Invasive lobular carcinoma	203	0.09 ± 0.19	
Other	107	0.30 ± 1.90	
**Tumor stage (*n* = 1087)**			
T1	279	0.18 ± 1.13	0.870
T2	631	0.14 ± 0.94	
T3	137	0.12 ± 0.31	
T4	40	0.11 ± 0.50	
**Nodal stage (n = 1070)**			
N0	514	0.19 ± 0.99	0.658
N1	360	0.14 ± 1.07	
N2	120	0.08 ± 0.40	
N3	76	0.11 ± 0.37	
**Distant metastatic status (*n* = 923**)			
No	901	0.17 ± 1.01	0.750
Yes	22	0.10 ± 0.25	
**ER status (*n* = 1040)**			
Negative	237	0.47 ± 1.74	<0.001
Positive	803	0.06 ± 0.48	
**PR status (*n* = 1037)**			
Negative	343	0.34 ± 1.40	<0.001
Positive	694	0.06 ± 0.60	
**HER2 status (*n* = 913)**			
Negative	702	0.17 ± 0.98	0.808
Equivocal or Indeterminate	26	0.10 ± 0.23	
Positive	185	0.13 ± 1.16	–

*ER, estrogen receptor; HER2, human epidermal growth factor receptor-2; N, node; PR, progesterone receptor; SD, standard deviation; T, tumor.*

**FIGURE 6:**
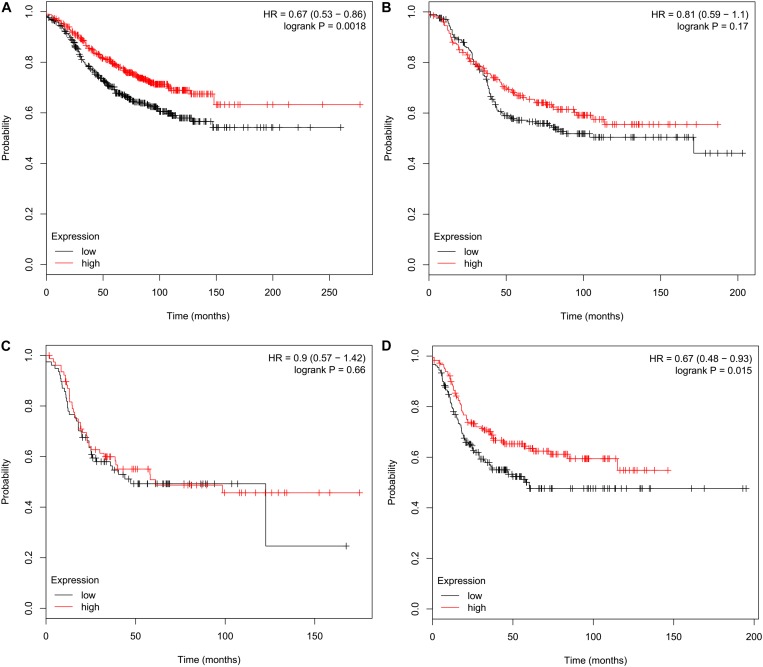
Effect of *CMTM5* mRNA expression levels on relapse-free survival in different breast cancer subtypes using the Kaplan–Meier plotter. **(A)** Luminal A, **(B)** luminal B, **(C)** *HER2*+, and **(D)** basal-like subtypes.

Relapse-free survival analyses using the KM plotter indicated that high *CMTM5* mRNA expression levels were related to better RFS in luminal A (HR 0.67, 95% CI 0.53–0.86, *P* = 0.0018) and basal-like (HR 0.67, 95% CI 0.48–0.93, *P* = 0.015) subtypes (Figures 6A,D); however, the mRNA expression levels of *CMTM5* were not associated with RFS at all in luminal B (*P* = 0.170) (Figure 6B) and *HER2*+ (*P* = 0.660) (Figure 6C) subtypes. Moreover, *CMTM5* mRNA expression levels did not correlate with RFS after stratification by tumor grade, nodal status, and *TP53* status.

**FIGURE 7:**
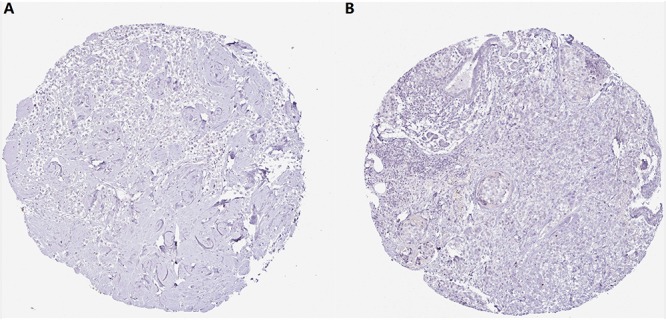
Representative immunohistochemical staining of *CMTM5* in breast cancer tissue samples from the Human Protein Atlas database (original magnification 50×). **(A)** Invasive ductal carcinoma (staining: not detected, intensity: negative, and quantity: none). **(B)** Invasive lobular carcinoma (staining: not detected, intensity: negative, and quantity: none).

With regard to the immunohistochemical staining of *CMTM5*, among the 11 breast cancer patients with pertinent data in the Human Protein Atlas, only one (9.1%) showed low expression of *CMTM5*, whereas no expression of *CMTM5* in tumor tissues was observed in the other 10 (90.9%) patients (Figures 7A,B).

### The Roles of *CMTM5* in Breast Cancer Therapies

**FIGURE 8:**
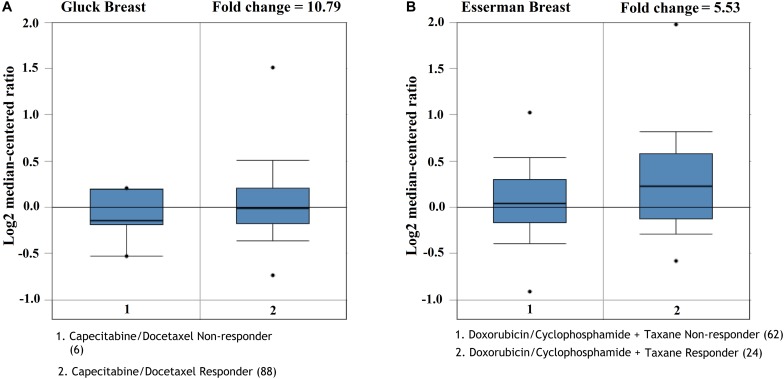
The influence of the expression of *CMTM5* on the response to chemotherapeutics in breast cancer (**A**, Gluck Breast database; **B**, Esserman Breast database).

We future analyzed the roles of *CMTM5* expression levels in response to breast cancer therapies using Oncomine. The results showed that lower expression of *CMTM5* was associated with lower response to systemic therapy compared to those with higher expression of *CMTM5* including the regimens of capecitabine/docetaxel (Gluck Breast database) and doxorubicin/cyclophosphamide + taxane (Esserman Breast database) (Figures 8A,B). These findings showed that lower expression of *CMTM5* might be related to inferior response of breast cancers to chemotherapeutics.

### Promoter Methylation Levels of *CMTM*5

**FIGURE 9:**
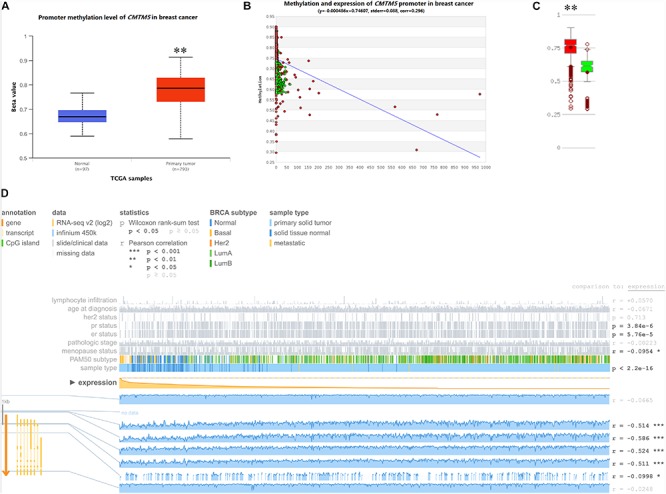
Impact of epigenetic alterations on *CMTM5* mRNA expression levels in breast cancer (^∗∗^*P* < 0.01). **(A)** Promoter methylation levels of *CMTM5* as per the analyses involving UALCAN. **(B,C)** Promoter methylation levels of *CMTM5* as per the analyses involving MethHC. **(D)** *CMTM5* expression levels in breast cancer related to DNA promoter methylation as per the analyses involving MEXPRESS.

DNA promoter methylation plays an important role in the epigenetic regulation of chromatin structure and gene transcription, and DNA promoter hypermethylation can induce stable epigenetic inhibition of gene expression. Herein we used UALCAN, MethHC, and MEXPRESS to investigate whether the dysregulated expression of *CMTM5* is related to DNA promoter methylation status. As per the analyses involving UALCAN, a significantly higher promoter methylation level of *CMTM5* was observed in breast cancer tissues than in normal tissues (1.17-fold, *P* < 1*e*−12) (Figure 9A), which was also confirmed upon using MethHC (*P* < 0.01) (Figures 9B,C). Moreover, MEXPRESS showed that the *CMTM5* mRNA expression level was negatively associated with DNA promoter methylation status (Figure 9D). The analyses involving MEXPRESS also indicated that the *CMTM5* mRNA expression level was significantly lower in breast cancer tissues than in normal tissues and correlated with *ER* (*P* = 5.76*e*−5), *PR* (*P* = 3.84*e*−6), and menopause status (*r* = −0.0954) (Figure 9D). These results suggested that DNA promoter hypermethylation of *CMTM5* negatively correlates with the mRNA expression level of *CMTM5* in breast cancer.

### *CMTM5*-Regulated Biomolecular Network

**FIGURE 10:**
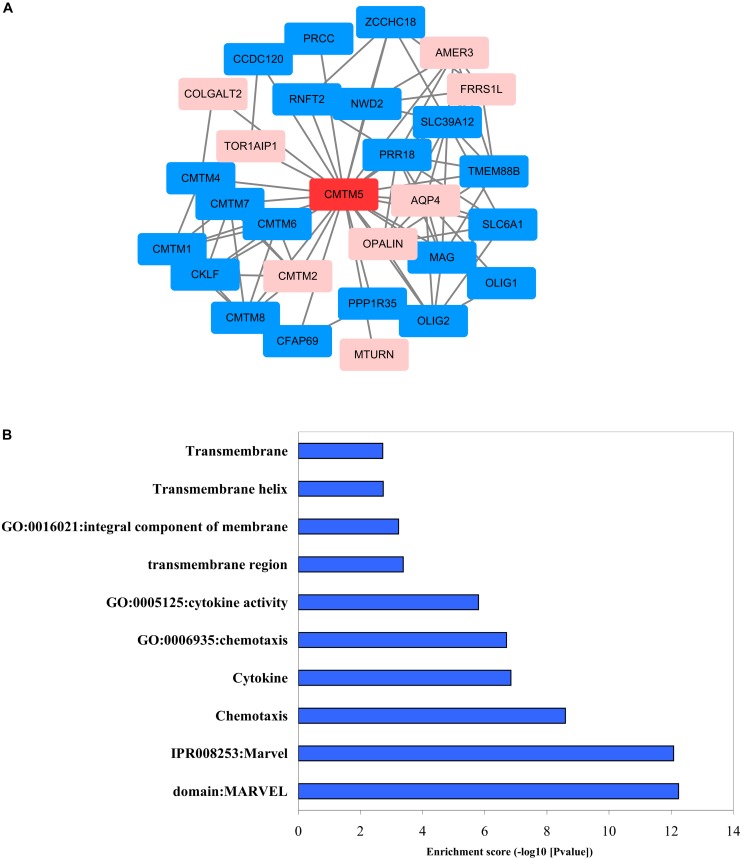
Biomolecular regulatory network of *CMTM5*. **(A)** Protein–protein interaction network complex and modular analysis using STRING (blue nodes indicate positive correlation and pink nodes show negative correlation). **(B)** Top 10 clusters identified via GO enrichment and KEGG pathway analysis using DAVID.

We used STRING and Cytoscape networks to assess the effects of regulatory genes on the carcinogenesis and progression of breast cancer. Functional interaction network analysis indicated that *CMTM5* is closely correlated with 28 genes including 20 genes were positive correlation with *CMTM5* and 8 genes were negative correlation with *CMTM5* (Figure 10A). We then performed GO enrichment and KEGG pathway analyses using DAVID; the obtained results showed that most significantly enriched gene sets related to MARVEL domains, chemotaxis, cytokines, transmembrane structures, and integral component of membrane (Figure 10B) were significantly enriched.

## Discussion

Herein we used Oncomine to analyze the expression of *CMTM* family members in various human cancers. We focused on breast cancer and *CMTM5*, and we report the results of a comprehensive bioinformatic analysis on the survival outcomes and potential molecular mechanisms using mRNA and protein expression levels, prognostic effects, genetic and epigenetic alterations, and corresponding biomolecular regulatory networks.

*CMTM* family members play a crucial role in the immune, male reproductive, and hematopoiesis systems (Lu et al., 2016); moreover, they serve as tumor suppressor genes and are associated with tumor progression in various human cancers (Bu et al., 2008; Li et al., 2011, 2015; Liu et al., 2013, 2015; Xie et al., 2014; Zhang et al., 2014, 2016; Gao et al., 2015; Xiao et al., 2015; Bei et al., 2017; Choi et al., 2018; Huang et al., 2019; Zhu et al., 2019). In fact a previous study even indicated that higher *CMTM1* and *CMTM3* expression levels are significantly associated with shorter overall survival in glioblastoma (Delic et al., 2015). In this study, we report that the mRNA expression levels of *CMTM1*, *CMTM2*, *CMTM4*, and *CMTM5* are significantly downregulated whereas those of *CMTM3*, *CMTM6*, and *CMTM8* are significantly upregulated in human cancers; therefore, our results indicate that the mRNA expression levels of *CMTM* family members can be highly divergent.

The dysfunction of *CMTMs* is closely correlated with the progression of numerous cancer types, and many of them may be strongly involved in the prognosis of patients with cancer; however, at present, little is known about the potential underlying mechanisms or their prognostic value in breast cancer. In addition to determining the mRNA expression levels of several *CMTM* family members, we herein used UALCAN and found that *CMTM5* was significantly dysregulated in breast cancer relative to other *CMTM* family members (45.36-fold vs. 1.02–3.72-fold). In addition, 11 breast cancer patients with available immunohistochemical staining of *CMTM5* in Human Protein Atlas, only 1 patient had low expression and 10 reflected no expression of *CMTM5*. Therefore, we chose *CMTM5* for further analyses.

Unlike other *CMTM* family members, *CMTM5* is localized separately on 14q11.2, a locus frequently altered in many types of cancers. *CMTM5* is widely expressed in normal tissues and its expression levels are either downregulated or silenced in liver (Guan et al., 2018), kidney (Cai et al., 2017), and prostate (Xiao et al., 2015) cancers. Shao et al. (2007) indicated that *CMTM5* exhibits tumor suppressor activities and is frequently silenced by methylation in carcinoma cell lines. They assessed the mRNA expression levels of *CMTM5* in breast cancer cell lines and reported undetectable or downregulated expression levels in all the cell lines. Moreover, *CMTM5* promoter methylation was observed in all downregulated or silenced breast cancer cell lines; a methylation-mediated mechanism was predicted to be involved in the development of breast cancer (Shao et al., 2007). To the best of our knowledge, this is the first study to confirm the downregulation of *CMTM5* mRNA and protein expression levels in breast cancer. Furthermore, we believe that promoter hypermethylation is a key reason for the inhibition of *CMTM5* mRNA expression and transcription.

In this study, we also found that lower expression of *CMTM5* was associated with inferior response to chemotherapeutics of breast cancers. *CMTM5* is located in 14q11.2, a chromosome region which have showed gains significantly more frequently in the chemoresistant disease subgroup compared to the chemosensitive disease subgroup in serous ovarian cancer (Kim et al., 2007). In addition, hypermethylator cancer cells also contribute to chemotherapy resistance of breast cancer (Sandhu et al., 2012). To the best of our knowledge, there were no studies assessing the role of *CMTM5* in chemotherapy sensitivity in human cancers. According to our study, *CMTM5* may be available as potential target for development of new treatment strategies of breast cancer.

Tumor heterogeneity refers to the difference in genes and phenotypes between cells of different tumors, it has been considered to be the biggest obstacle to the clinical management of breast cancer. In addition, heterogeneity is a fundamental and key element in cancer initiation, progression, and distant metastasis (Turashvili and Brogi, 2017). In our study, we found that patients with *ER* and *PR* positive diseases had lower levels of *CMTM5* expression. However, low mRNA levels of *CMTM5* were associated with lower RFS in our study, while *ER* and *PR* positive diseases were associated with better survival outcomes of breast cancer (Untch et al., 2019). Moreover, our study found that lower levels of *CMTM5* expression were related to lower RFS in luminal A and basal-like subtypes, but not in luminal B and *HER2*+ subtype, which indicated that this was also present with tumor heterogeneity in the *CMTM5* expression. The magnitude of the influence of tumor heterogeneity on *CMTM5* expression and its clinical significance still need further exploration.

By browsing biomolecular regulatory networks for genes of interest and via functional enrichment analyses, we found that *CMTM5* is closely correlated with 28 genes that are involved in pathways that potentially play a crucial role in breast cancer development and progression. For example, as anticipated, genes related to MARVEL domains, transmembrane structures, and chemokines were significantly enriched. The product encoded by *CMTM5* is closer to *TM4SF* (Han et al., 2003). *TM4SF* includes several types of proteins possessing the four transmembrane-helix structure, such as the classical TM4SF (tetraspanin) and MARVEL-domain containing proteins, and such proteins have been linked to breast cancer development and progression (Bouchagier et al., 2014; Jiang et al., 2019; Lara-Lemus, 2019). *CMTM5* also encodes a protein that potentially has a four transmembrane-helix architecture, which provides a theoretical basis for the new tumor suppressor gene of *CMTM5*.

Chemotaxis has a key role in cancer progression (Roussos et al., 2011). Some recent studies have reported that chemotaxis occurs during metastasis in many types of cancers, including breast cancers (Wang et al., 2004; Friedl and Alexander, 2011; Roussos et al., 2011). Several upregulated chemokines in human cancers are reportedly prominent tumor promoters (El-Haibi et al., 2013; Singh et al., 2016). Chemokines can generate a tumor immunosuppressive microenvironment and support cancer cells in their metastatic journey toward secondary tumors (Singh et al., 2011). In breast cancer, chemokines are mainly involved in the generation of a protumoral microenvironment and organ-directed metastasis; moreover, they mediate disease progression and contribute to the growth and proliferation of tumor cells (Palacios-Arreola et al., 2014).

Another important pathway that was identified to be associated with *CMTM5* was the negative regulation of cytokines. Cytokines are low molecular weight proteins that are involved in cell-to-cell communication by autocrine or paracrine signaling. Cancer cells communicate with the host mainly through cytokines and use this system for shaping the tumor microenvironment, promote tumor dissemination, and for epithelial–mesenchymal transition, motility, and invasion (Yamaguchi and Condeelis, 2007). In addition, inflammatory cytokines play a role in the invasiveness and migration of breast cancer cells (Jorcyk et al., 2006; Bolin et al., 2012), and they are also implicated in the differentiation, apoptosis, angiogenesis, and survival of breast cancer cells (Scheller et al., 2006, 2011). Moreover, within the sites of metastasis, distinct molecules, such as cytokines and chemokines, are involved in attracting tumor cells that express the corresponding cell surface receptors (Ben-Baruch, 2003).

Tawara et al. (2019) reported that a high expression of cytokine or cytokine receptors in breast cancer was associated with lower survival. Moreover, patients with high expression of cytokines showed lower survival than those with low expression of cytokines in *HER2−*, but not in *HER2*+, disease (Tawara et al., 2019). Our study also confirmed that high mRNA expression levels of *CMTM5* were associated with better RFS in luminal A and basal-like subtypes (*HER2−* disease) and not associated to RFS at all in luminal B and *HER2*+ subtypes (*HER2*+ disease). An earlier study proposed that interleukin-6 family cytokines can be used as profiling biomarkers for *HER2−* breast cancer tissues, particularly in cases of triple-negative breast cancer, but they had no significant association with *HER2*+ cancer tissues (Niu et al., 2002). Therefore, these results suggest the presence of redundancy between *CMTM5-* and *HER2*-induced signaling.

One limitation of this study is that most analyses involved determining mRNA expression levels; further studies should thus be conducted to validate our results at a protein level by performing, for example, Western blotting. Although our study highlights the prognostic value of *CMTM5* in breast cancer and predicts several potential pathways implicated in its function, more studies are warranted so as to elucidate the specific roles of *CMTM5* and underlying mechanisms that affect breast cancer development and progression.

## Conclusion

In conclusion, using comprehensive bioinformatic analysis, we report that *CMTM5* is a putative tumor suppressor gene that can serve as a potential negative prognostic biomarker in breast cancer. We believe that our findings should be helpful for investigating the detailed function and potential carcinogenic mechanisms of *CMTM5* in breast cancer, which might promote the development of a novel target treatment for breast cancer.

## Data Availability Statement

Publicly available datasets were analyzed in this study. This data can be found here: www.cbioportal.org, http://www.oncomine.org, http://ualcan.path.uab.edu, http://kmplot.com/analysis/index.php?p=service&cancer=breast, http://string-db.org, and https://www.proteinatlas.org.

## Author Contributions

JZ, JW, S-GW, and Z-YH are lead authors who participated in data collection, manuscript drafting, table and figures creation, and manuscript revision. C-LL, LH, and JL aided in data collection. JZ, JL, and JW are senior authors who aided in drafting the manuscript and manuscript revision. Z-YH and S-GW are the corresponding authors who initially developed the concept and drafted and revised the manuscript. All authors read and approved the final manuscript.

## Conflict of Interest

The authors declare that the research was conducted in the absence of any commercial or financial relationships that could be construed as a potential conflict of interest.

## Footnotes

1 www.cbioportal.org

2 http://www.oncomine.org

3 http://ualcan.path.uab.edu

4 http://kmplot.com/analysis/index.php?p=service&cancer=breast

5 http://string-db.org

6 https://www.proteinatlas.org
